# *In Silico* Experimental Modeling of Cancer Treatment

**DOI:** 10.5402/2012/828701

**Published:** 2012-02-01

**Authors:** Trisilowati, D. G. Mallet

**Affiliations:** ^1^Mathematical Sciences Discipline, Queensland University of Technology, P.O. Box 2434, Brisbane, QLD 4001, Australia; ^2^Institute of Health and Biomedical Innovation, Queensland University of Technology, P.O. Box 2434, Brisbane, QLD 4001, Australia

## Abstract

*In silico* experimental modeling of cancer involves combining findings from biological literature with computer-based models of biological systems in order to conduct investigations of hypotheses entirely in the computer laboratory. In this paper, we discuss the use of *in silico* modeling as a precursor to traditional clinical and laboratory research, allowing researchers to refine their experimental programs with an aim to reducing costs and increasing research efficiency. We explain the methodology of *in silico* experimental trials before providing an example of *in silico* modeling from the biomathematical literature with a view to promoting more widespread use and understanding of this research strategy.

## 1. Introduction

Traditional laboratory-based cancer research involves expensive trial and error experimental strategies applied to humans, animals, and their harvested tissues. “*In silico* experimentation,” the coupling of current computing technologies with mathematical or theoretical characterizations of cancer cell biology, provides a novel approach to guiding the early stages of hypothesis development and experimental design that has the potential to create subsequent efficiencies and cost savings in the laboratory. This computational approach is advantageous because it allows vast numbers of experiments to be carried out that are easily observed at any desired level of detail and can be repeated and controlled at will.

It seems difficult to argue that preclinical studies in cancer biology are expensive. Such studies involving in vitro and in vivo animal experiments involve hypothesis generation and testing to determine whether further trials are warranted and are extremely costly both in terms of researchers' time and the associated financial investment. Costs, such as laboratory setup, equipment and space, time spent by academics training others, and the time, equipment, and materials costs involved in repetitive, hands-on experimental work, all contribute to the expense of laboratory-based experimental research.

Our contention in this paper, a view shared by many researchers in the closely related fields of computational, theoretical and mathematical biology, is that *in silico* experiments can be used as precursors to, or in combination with, preclinical experimental studies to provide guidance for the development of more refined hypotheses and experimental studies. *In silico* and mathematical modeling lends itself to the determination of preliminary information such as toxicity, pharmacokinetics, and efficacy, which can then be used to guide preclinical and clinical studies.

*In silico* experimentation involves the combination of biological data and expert opinion with mathematical and computer-based representations to construct models of biology. Computer-based experiments can then be carried out using these models rather than, or in combination with, laboratory research. Using parameter distributions based on current expert opinion (“fuzzy” inputs) or actual biological data (random variables) as inputs into the *in silico* models, it is possible to create what are effectively “computational patients” upon which to experiment. It is of course also possible to consider smaller-scale experiments and even multiscale experiments, conducted on molecular, cellular, and tissue/organ levels. Appropriate use of *in silico* models involves making predictions based on experimental data and expert information and allows the models to be effectively used to inform clinical trials with a view to reducing costs and increasing efficiency.

To provide an example, consider the study of cell transfer therapy for metastatic melanoma patients of Rosenberg et al. [1]. The authors commented on the difficulty of deriving meaningful results from human experiments because of the variations in cell types, tumor types, immune states, and more fundamentally the human subjects themselves. While Rosenberg et al. suggest a solution to such a problem is to treat the same patient in differing ways over a period of time, another more ethical and flexible, and less hazardous method is through the use of *in silico* models and experimentation. This approach was used in the model discussed in Section 3. 

There is a rich history of theoretical studies involving mathematical and computational approaches to studying cancer. Burton and Greenspan pioneered the mathematical modeling of tumor growth with models of growth dynamics explained as a problem of diffusion [2–5]. Since that time, theoretical studies of most aspects of tumor growth and related processes have been investigated at least to some extent, using various different methodologies including differential equations, stochastic models, and cellular automata. Araujo and McElwain provide an excellent review of the mathematical modeling work carried out up to middle of the last decade [6]. More recently, Alarcón et al. [7], Mallet and coworkers [8, 9], and Ferreira et al. [10, 11] have used a new paradigm—that of spatiotemporal, stochastic models using hybrid cellular automata techniques—to represent “computational patients” or “*in silico* experiments” in a new direction for cancer research. This experimental paradigm extends the traditional mathematical modeling of cancer to incorporate computational simulations that are parameterized in such a way to represent different patients or different experiments.

It is also becoming more common to find mathematical studies appearing in the cancer literature. Utley et al., for example, discuss improvement in survival rates resulting from postoperative chemotherapy for lung cancer patients [12]. They note that the marginal (5%) survival rate improvement due to chemotherapy may be outweighed for some patients by the morbidity due to the treatment and that further trials do not actually improve information provided to patients, but rather improve the certainty of that prediction. Utley et al. propose the use of a mathematical model, utilizing patient-specific pathological cancer stage data combined with existing techniques, to arrive at better evidence for informing patients regarding their postoperative treatment choices. 

In a study more at the preclinical stage of research, de Pillis et al. describe a differential equation-based model for the interactions between a growing tumor, natural killer cells, and CD8^+^ T cells of the host immune system [13]. With a view to understanding how the immune system assists in rejecting growing tumors, de Pillis et al. present mathematical descriptions of key mechanisms in the immune response before fitting the model to data from published mouse and human studies. A parameter sensitivity analysis reveals the key role of a patient-specific variable and that the model may in fact provide a means to predict positive response of particular patients to treatment.

Mallet and de Pillis [8] and later de Pillis et al. [9] explored a particular type of *in silico* model known as a hybrid cellular automata-partial differential equation (CA-PDE) model to describe the interactions between a growing tumor and the host immune response. A hybrid CA-PDE model combines the traditional continuum methods of applied mathematics, such as macroscale reaction-diffusion equations describing chemical concentrations, with more modern, individual, or grid-based automaton methods, which are used for describing individual cell-level phenomena. The hybrid CA-PDE modeling approach has been successfully used in the past to model tumor growth, chemotherapeutic treatment, and the effects of vascularization on a growing tumor [7, 10, 11, 14]. In Section 3 we discuss this model in some detail, explaining how the model is constructed as well as typical outputs of an *in silico* model of this type.

## 2. Methods—*In Silico* Trials

While in vitro and in vivo models use actual biological materials and/or actual animals to investigate hypotheses and, for example, predict effectiveness of treatment strategies, *in silico* models use specifically designed computer programs to mimic these “real” experimental environments and to conduct computational experiments. There exist a number of different types of *in silico* model including differential equation models that track changes in quantities over time and/or space, network models that trace lines of probabilistic causation and/or correlation, discrete cellular automata- or individual-based models, and hybrids of all of these models. Rather than providing models of real biological phenomena and structures that have a basis in some sort of extracted tissue or a somehow related animal species, these *in silico* models are comprised of mathematical and computational representations such as formulae, equations, and/or computer programs. A key feature of such models is that they can be “parameterized” so that quantities or rates not known in the real world or which are specific to different experiments can be investigated via computational experiments, or as we dub them “*in silico* trials.” The concept of the *in silico* trial can be thought of as akin to clinical trials. Just as each patient in a clinical trial has their own set of characteristics such as height, age, and status with regard to smoking and alcohol consumption so too we can run the program of an *in silico* model multiple times with varied parameters to produce “computational patients” in an *in silico* trial. 

The development of *in silico* model is often a process of cross-disciplinary collaboration between cancer biologists and mathematicians or modelers. Generally, the initial stages involve the model builder obtaining an understanding of the tumor biology required for developing the *in silico* model. This will be a period of intense collaborative work involving discussions between all investigators and a review of the theoretical and experimental literature. The next stage involves abstraction of biological information into a mathematical or computational form, that is, building the update rules. This requires the creation of mathematical representations of relevant micro level biological phenomena and mechanisms (such as rates and results of cell division, methods for representing distributions of chemical molecules, and interactions between antigen and antigen presenting cells) and the compilation of these into a macrolevel description of the real experimental situation.

Following the development of the update rules, the algorithm for the entire process is computerized usually employing generic programming languages such as C++ or with mathematical software such as MATLAB. This algorithm allows for the solution of the *in silico* model and facilitates easy simulation of large numbers of experiments, that is, repeated simulation of the model using many different parameter sets in order to mimic running slightly different experiments in the laboratory. This could reflect, for example, an investigation of the effect of different quantities of gold nanoparticles on effectiveness of radiotherapy or the effect of different concentrations of chemotherapeutic treatments.

While largely automated via the computer program, the simulation of the *in silico* model requires careful and continuous monitoring to ensure that computations converge (i.e., solutions are obtained rather than computational errors) and to make adjustments to investigations when results of interest are observed.

Following simulation of the *in silico* model, the results of the computational experiments are analyzed and interpreted. This generally involves the use of custom-designed visualization of the resulting data. The investigators use the outputs of the model to determine what results are already useful for informing any associated experimental studies as well as what parts of the *in silico* model are deficient and require refinement along with a follow-up round of *in silico* experiments. The whole process can be repeated, with refinement, as often as new information is required, and in general the costs of follow-up *in silico* experimentation decrease as the fundamental computational framework has already been developed. In the remainder of this section, we present an oversimplified and generic model along with the computational algorithm to further illuminate this concept.

### 2.1. The CA Approach

**Figure 1:**
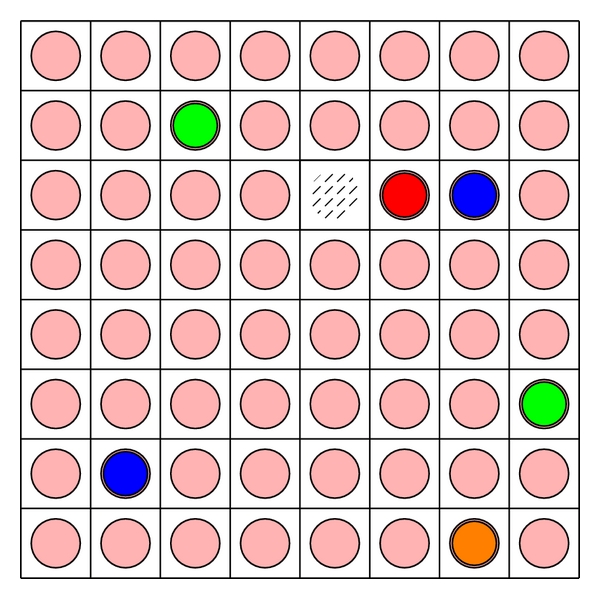
A two-dimensional grid is imposed on a region of space of interest with cells of different types, molecules, debris, fluid, and/or bacteria housed in each element of the grid.

**Figure 2:**
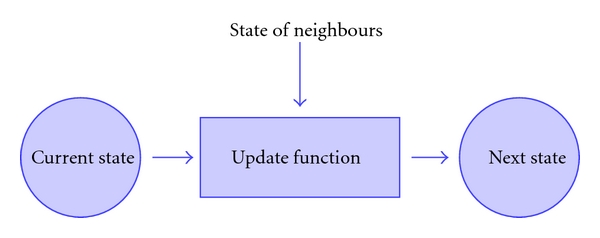
The transition from the current state to the next state for each element of the CA grid is determined only by its current state, that of its neighbors and the update rule.

A cellular automaton (CA) is a type of mathematical model, discrete in both space and time. Here we consider a two-dimensional CA, such as that which could be used to model the surface of the skin or possibly a petri dish, but note that three-dimensional models are simple, if computationally expensive, extensions of the same concepts. A two-dimensional CA consists of a lattice or grid of CA elements covering a region of space (see Figure 1). Applied in the biological context, each element is allowed to house one or more biological cells and, depending on the experimental situation being modeled, may also hold other matter such as molecules, debris, fluid, or bacteria. The cells in the CA elements are allowed to interact with one another via update rules. The set of update rules defines how the state of each element changes in response to its current state and the current state of its neighbors—the definition of these rules is the fundamental modelling stage in the development of the *in silico* model (see Figure 2). The accuracy of the model is heavily dependent on designing rules that adequately reflect the real interactions between cells.

The type of cellular automata model considered here is executed as follows. The system is first initialized so that the computational representation presented in the cellular automata grid matches some initial condition for the ensuing computational experiment. Next, a sequence of “time steps” is carried out such that the model time is incremented by a small amount at each step. Within each time step, every spatial location or element in the CA grid is investigated to identify its contents. Depending on the contents, an appropriate update rule is applied which may involve the states of the neighboring elements. Updates are made throughout the grid, time is incremented, and the process continues.

To extend this model to allow *in silico* trials, the computer program for the algorithm described in the above paragraph is wrapped in a further program. This involves providing a collection of two or more (depending on the number of experiments or trials required) parameter value sets to the algorithm and running the algorithm once with each set. The output data, for example, cell counts over time, for each trial is exported to memory at the completion of each trial.

### 2.2. Development of Rules

As mentioned earlier, with regard to developing an accurate description of the biological process of interest, the specification of the update rules for a cellular automata-based *in silico* model is the most important part of the modelling process. To demonstrate this, consider the seemingly simple case of the movement of one cell to a neighboring location and the following increasingly complex but increasingly accurate rules.

**Rule 1**

If there is one or more empty CA elements surrounding a cell, move to a randomly chosen empty element, otherwise, do not move.

**Rule 2**

If there is one or more empty CA elements surrounding a cell and moving to one would increase the cell's satisfaction in some way, move to a randomly chosen element of this type, otherwise, do not move.

**Rule 3**

If there is one or more empty CA elements surrounding a cell, consider moving to one of these locations with a probability that depends on factors such as cell adhesion levels, nutrient supply, and chemoattractants, otherwise, do not move.

Each of these rules could be implemented in an *in silico* model as the determining factor regarding whether or not a cell moves. Clearly moving from Rule 1 to Rule 3, the amount of realism increases, but, simultaneously, the amount of information required to design the rule also increases. Rule 1 does not require any information about the cells of interest—the cell simply moves if it can, and the location it moves to is randomly chosen. On the other hand, Rule 3 requires that the modeler has some preexisting or obtainable understanding regarding how cells respond to chemoattractants, how cell adhesion affects motility, and what impact nutrient levels have on the decision of a cell to move from location to location. Thus we note that with more information about the biological process, the modeler can construct more realistic update rules, but at the same time, a lack of information by no means rules out *in silico* modeling. In fact, *in silico* models can yield rich information when they are used from the very early stages as part of hypothesis generation and testing when there is a dearth of biological information.

## 3. An Example in Cancer Biology

Mallet and de Pillis [8] presented a so-called “hybrid cellular automata model” of the interactions between the cells of a growing tumor and those of the host immune system. Mallet and de Pillis successfully designed a computational method for investigating the interactions between an idealized host immune system and a growing tumor. The simulated tumor growth experiments were found to be in qualitative agreement with both the experimental and theoretical literature. It was found that even with quite simple mathematical descriptions of the biological processes and with an overly simplified description of the host immune system, the computational model had the potential to produce the behavior observed in laboratory experiments including spherical and papillary tumor growth geometries, stable and oscillatory tumor growth dynamics, and the infiltration of the tumor by immune cells. It was also possible to show the dependence of these different morphologies on key model parameters related to the immune response. Numerical solutions produced using the Mallet and de Pillis model agreed qualitatively with the experimental results demonstrated by Zhang et al. [15], Schmollinger et al. [16], and Soiffer et al. [17].

While a laboratory model is usually designed to focus on a particular stage of a process or a specific event, *in silico* models can be designed to focus on arbitrarily small or large-scale phenomena. Mallet and de Pillis chose to focus on the early stages of tumor growth during which the tumor is adjacent to, but not yet infiltrated by, nutrient supplying vasculature in order to allow for an investigation of the initial interactions between the immune system and the emerging tumor. The simple model incorporated a simplified immune system comprised of two cell types, namely, the natural killer (NK) cells of the innate immune system and the cytotoxic T lymphocytes (CTLs) of the specific immune system. A hybrid cellular automata and partial differential equation model was constructed with an aim to demonstrate the combined effects of the innate and specific immune systems on the growth of a two-dimensional representation of a growing tumor. This was accomplished by constructing a model with computerized cell behaviors built from descriptions in the experimental literature and findings of dynamic models of tumor—immune system interactions developed in the theoretical literature such as the work of Kuznetsov and Knott [18] and de Pillis and Radunskaya [19, 20].

Mallet and de Pillis' hybrid cellular automata model employed a coupled deterministic-stochastic approach that had the benefit of being conceptually accessible as well as computationally straightforward to implement. The authors used reaction-diffusion equations, to describe chemical species such as growth nutrients, and a cellular automata strategy to track the tumor cells and two distinct immune cell species. Together, these elements simulated the growth of the tumor and the interactions of the immune cells with the tumor growth. 

The model tracked cells both through time and through space—a clear advantage over dynamic models that assume a spatially well-mixed population of cells, which is not often the case in reality. Unlike continuum-based spatiotemporal models, which are generally comprised entirely of partial differential equations, the hybrid cellular automata approach allows for the consideration of individual cell behavior and associated randomness, rather than applying a general rule to a collection of cells, as is the case with continuum models. The cellular automata approach is also very flexible in terms of its computational implementation. While the Mallet and de Pillis model considered only four cell species with an overly simplistic view of the immune system, it is easily modified to cater for the inclusion of more cell types or new chemical species.

The evolution of the cell species considered in the Mallet and de Pillis model proceeds according to a combination of probabilistic and deterministic rules, developed in an attempt to describe the phenomena considered important in the theoretical model. In particular, Mallet and de Pillis imposed a simplifying assumption to the host cells such that, other than their consumption of nutrients, they allow tumor cells to freely divide and migrate and were more or less passive bystanders to tumor growth. Tumor cells on the other hand were able to move, divide, die due to nutrient levels and die because of the immune response, each with a probability that depended on some combination of nutrient levels, local immune response, and crowding due to the presence of other tumor cells. Natural killer cells were maintained at or near a “normal” level by recruitment from outside the domain of interest whenever the local density dropped too far below its equilibrium level. Both natural killer cells and cytotoxic T cells were able to lyse tumor cells, although CTLs could do so more than once and were able to attract other CTLs to the local area. CTLs were also subject to removal from the local region with a probability depending on the local tumor cell density.

The rules used to represent these phenomena are developed as approximations of reality and involve considering individual events, such as an interaction between a cell on the periphery of a tumor and a natural killer cell, and attempting to quantify what happens as a result of this interaction. This act of quantifying is guided by accepted results in the experimental and theoretical literature, expert elicitation, and simple physical arguments. As mentioned in the previous section, the development of these rules is the most important step in model development.

While the design and statement of all the CA rules are presented in the original paper, here we expand on the design of one of the rules to elucidate how such objects are constructed. Take, for example, the individual cell level event of cell division. This process is extremely complex and involves countless subprocesses each with many participants. Just as an experimentalist in the laboratory does not consider each of these explicitly, we do not attempt to represent each of them in the computational model either. Instead, we distil what information is available in the literature and from collaborators to arrive at a model representation of the chance that the event occurs given certain conditions. This distilled model representation is the cellular automata rule.

For the case of cell division, Mallet and de Pillis consider that given a tumor cell, the probability of division increases with the ratio of nutrient concentration to the number of tumor cells already present in the local region. Note that there is no mention of subcellular signal processing and neither is there any consideration of macrolevel pressure fields. Instead, the chance of the occurrence of a cell division is condensed into a consideration of whether or not there are sufficient nutrients nearby and whether or not the region is already crowded with tumor cells.

This rule is interesting because it also incorporates a second subrule—that of the placement of the daughter cell. The model dictates that the grid location upon which the daughter cell is placed depends upon the cells occupying the neighborhood of the mother cell. For example, a dividing cell with at least one host cell or necrotic space surrounding it will place its daughter cell randomly in one of those noncancerous locations and either destroy the host cell or simply replace the necrotic material. On the other hand, if all elements around the dividing cell are filled with tumor cells, the daughter cell will be placed in the neighboring element containing the fewest tumor cells. The authors viewed this as one approach to modeling tumor cell crowding.

**Figure 3:**
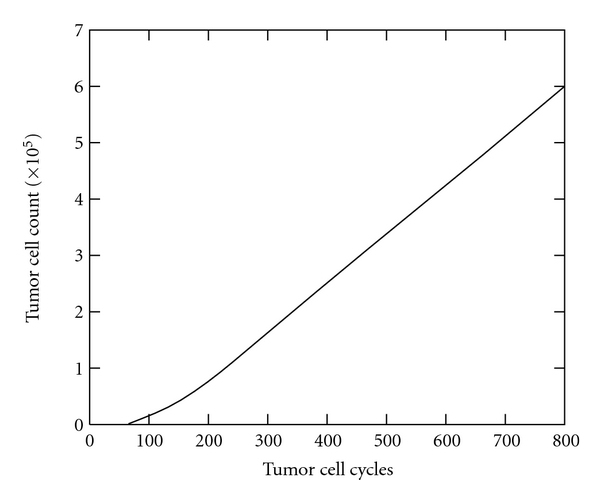
An example of growth curve produced by the Mallet and de Pillis *in silico* model showing total number of tumor cells over time for a tumor growing in the absence of immune response.

**Figure 4:**
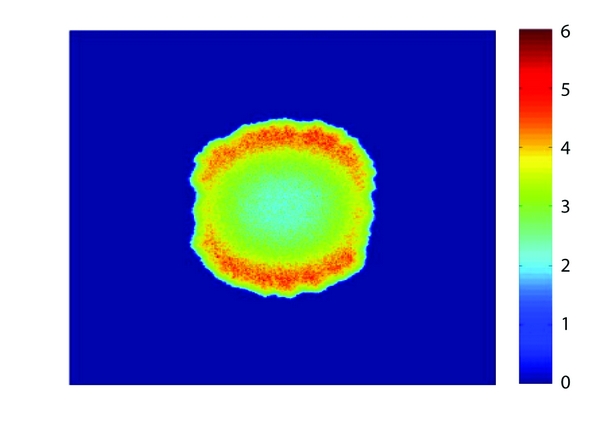
An example of two-dimensional tumor growth after 800 cell cycles, simulated using the Mallet and de Pillis *in silico* model. Red intensity indicates tumor cell density. The domain shown is approximately 10–20 mm square, and growth is over a time period of at least a year.

*In silico* models such as that of Mallet and de Pillis can produce an array of different outputs. In this particular work, the authors focused on presenting growth curves and two-dimensional spatial snapshots in time of growing tumors that were compared with experimental results. Figures 3 and 4, for example, show a growth curve and two-dimensional snapshot of a tumor growing in the absence of the immune system. This result was used as a baseline to compare with experimental and previous mathematical results prior to investigating the effects of the immune system with this new model. Note, in Figure 3, the initially exponential growth phase (cycle 0–200), before a phase of linear growth (cycle 200–800). These growth characteristics mimic the growth rates described in the experimental work of Folkman and Hochberg [21] and mathematically by Greenspan [3].  Figure 4 is a snapshot in time (800 cell cycles) of the same simulation where we see a roughly circular tumor with a radius of about 200 cells growing steadily outward toward the sources of the nutrient. Higher tumor cell densities are seen at the periphery of the tumor while in the center, a necrotic core is beginning to form with some necrotic material already appearing. 

**Figure 5:**
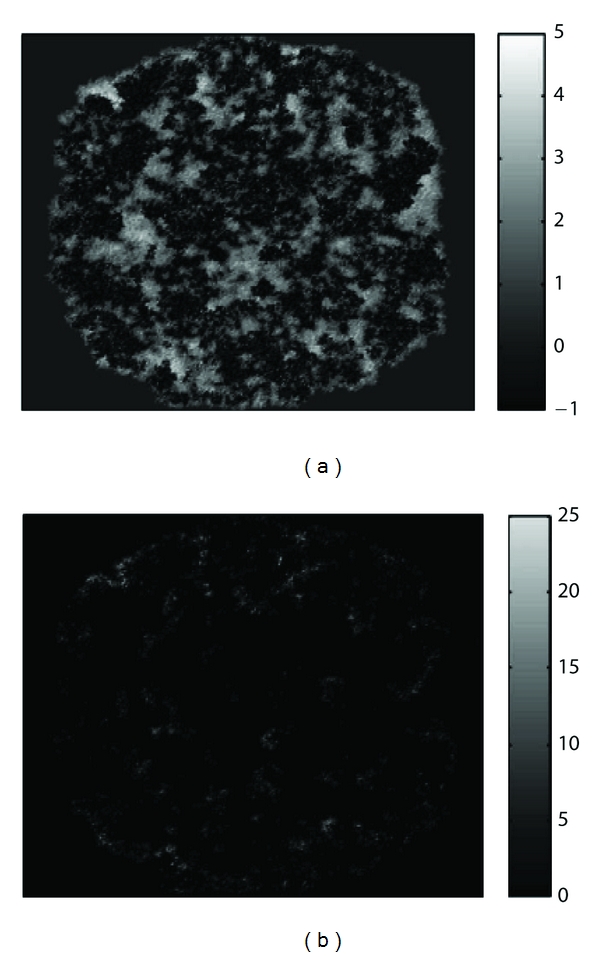
Two-dimensional snapshots of a tumor exhibiting high levels of necrosis (a) and populations of immune cells that have infiltrated the tumor mass causing cell death (b).

Mallet and de Pillis also presented a particularly interesting application of their model that produced qualitatively similar simulated tumors to the results of some recent experimental studies of immune response to tumor growth. The experimental studies of Schmollinger et al. [16], Soiffer et al. [17], and Kuznetsov and Knott [18] discussed the relationship between increased survival rates of cancer patients, tumor necrosis, and fibrosis, and the presence of intratumoral T cells or infiltrated T lymphocytes. In Figures 5(a) and 5(b), immune cells are shown to have infiltrated a growing tumor. In particular, the darker regions in Figure 5(a) are evidence of tumor necrosis while lighter regions of Figure 5(b) are indicative of high immune cell populations. These solution plots are similar to experimental results shown by Schmollinger et al. [16], Soiffer et al. [17], and Kuznetsov and Knott [18] where strings of immune cells are moving into the tumor, surrounding individual cells, and causing tumor cell necrosis.

The simulation results showed employee parameters for a compact tumor (in the absence of the immune system), low-level CTL recruitment, and low CTL death probability. We emphasize again that the same computer program is used to implement these simulations as those considered in the previous figures; varying system parameters is all that is required to consider quite a different experiment when using the *in silico* modeling technology.

The example of an *in silico* model presented in this section employed a moderately complex, hybrid cellular automata-partial differential equation methodology to describe interactions between the host immune system and a growing tumor. In the absence of a simulated immune system, the model was capable of reproducing both compact-circular and wild papillary tumor morphologies. Morphology change was directly related to the relative rates of consumption of the survival and mitosis nutrients by both tumor and host tissue cells, and the results presented correspond qualitatively with the experimental literature (such as Folkman and Hochberg [21]). When the model allowed for a simulated immune system, with different choices of T-lymphocyte recruitment and/or death parameters, oscillatory growth curves were observed for nearly all parameter sets. Depending on the strength of the immune system recruitment and death parameters, the tumor growth either increased without bound or resulted in destruction of the invasive growth. The model was also able to reproduce experimentally observed immune cell infiltration of growing tumors.

The different sets of parameter values used in the simulation of the Mallet and de Pillis model are the primary method for computationally mimicking different strengths of immune systems of, for example, healthy individuals, capable of early tumor detection and destruction, and individuals in poor immune health, for whom tumors grow easily. In summary, even though the update rules proposed in the Mallet and de Pillis model were relatively simple and the number of cell types considered was far from exhaustive, the authors created an *in silico* model that was able to produce results in qualitative agreement with both the experimental and theoretical literature and which could be improved upon to provide useful preclinical results of relevance for further model development for guiding experimental work related to various treatment and vaccination strategies.

## 4. Conclusions

*In silico* experimental modeling of cancer involves combining findings from biological literature with computer-based models of biological systems in order to conduct investigations of hypotheses entirely in the computer laboratory. In this paper we have presented a discussion of the concept of *in silico* modeling and how *in silico* models are constructed in practice. We have presented an example of *in silico* modeling that is relevant to the study of cancer and discussed its application and use as a hypothesis-generating tool as a precursor to or in combination with traditional clinical and laboratory research. This type of computational tool, when used in transdisciplinary research teams, has the potential to allow researchers to refine their experimental programs with an aim to reducing costs and increasing research efficiency, and we advocate increased use of such strategies by research groups.
